# Alcohol and Betting Radio Advertising in Spain. A Comparative Analysis of the Minor’s Protection Time Slot from a Media Responsibility Perspective

**DOI:** 10.3390/ijerph17238873

**Published:** 2020-11-29

**Authors:** Salvador Perelló-Oliver, Clara Muela-Molina, Luis M. Romero-Rodríguez

**Affiliations:** 1 Department of Communication Sciences and Sociology, Universidad Rey Juan Carlos, 28492 Madrid, Spain; salvador.perello@urjc.es (S.P.-O.); clara.muela@urjc.es (C.M.-M.); 2 ESAI Business School, Universidad de Especialidades Espítiru Santo, Guayaquil 092301, Ecuador

**Keywords:** radio mentions, radio advertisement, media responsibility, betting advertisement, alcohol advertisement, health communication, media self-control, media regulations, protected time-slots, consumer behavior

## Abstract

This research analyzes the presence of advertising and radio mentions of alcoholic beverages and sports betting, two products that represent harmful behaviors for the audiences’ health. To do this, a quantitative content analysis was undertaken for all of the programming of the three most listened radio stations in Spain (Cadena Ser, COPE, and Onda Cero) throughout 2017, obtaining a total corpus of 2848 radio messages distributed as follows: 266 radio spots and 2582 radio mentions. The messages were also analyzed according to the broadcasting schedules (protected time-slot or non-protected time-slot). The results showed that advertising and sports betting mentions were more present in the regular programming of the three stations (*n* = 2304), with mentions (*n* = 2582) being more numerous than advertising spots (*n* = 544). Moreover, it is evident that in practice, none of the radio stations respected the protected time slots since the majority of the mentions and spots of high alcoholic beverages and sports bets were verified between 6:00–21:59 (*n* = 2522). These results show the prevailing need for greater control over this type of content by public entities, demonstrating a significant lack of regulation by the media’s self-control mechanisms.

## 1. Introduction

### 1.1. Alcohol Consumption in Spain

Strongly rooted in today’s societies, alcohol consumption is a massive social and economic burden and is one of the leading causes of addiction, morbidity, and mortality in the world, especially in adults, leading to the premature death of more than three million people per year [1]. In the case of Spain, 91.2% of Spanish people have consumed alcohol at least once in their lives, with an average age of onset of consumption of 16.6 years old, while at least 75.2% of Spanish people claim to have consumed some type of alcoholic beverage in the last year [2]. These data put into context that by 2018, 3.5 million people in Spain consumed alcohol more than once a day, twice as much as in 2017 (1.72), a pattern that is replicated by those who consume it between three and four times a week [3]. 

On the other hand, daily alcohol consumption increases with age: in the population between 45 and 54 years old, 17% consumed alcohol daily, increasing by 8 points in the age group between 55 and 64, and another three points in the age group between 65 and 74 (with a final total of 28%). However, social consumption, defined by socialization patterns, reverses the results, with age ranges between 25 and 34 (30%) and 35 to 44 (28%) being the ones with the highest intake [4]. For high alcoholic drinks, also called spirits, their sales volume reached 213.2 million liters in 2019, which meant an income of 8.8 million euros and an average consumption per capita of 4.6 L [5], placing Spain as the fifth-highest country in Europe for consumption, while the alcoholic drinks industry contributes to the public treasury approximately 1.4 million euros per year [6].

It is worth noting the crucial importance of this sector in the Spanish economy in terms of its contribution to the GDP, jobs, and tax revenues for public finances and, therefore, the reluctance of the industry to limit the main centrifugal force of a demand encouraged by the 21.06 million euros of advertising investment in high alcohol content beverages in 2018 [7]. 

Alcohol consumption is one of the significant public health problems to be addressed, being the cause of neuropsychiatric and behavioral disorders as well as severe trauma and causing deaths in traffic accidents associated with its abuse, in addition to being the trigger for more than 200 diseases, some of them serious such as liver cirrhosis or some types of cancer [1]. In this sense, alcohol was the cause of 17.6% of emergency room admissions in Spain in 2019, totaling 8662 cases for all age groups, of which almost 7000 were among adult consumers between 35 and 64 years of age [8]. Dramatically, alcohol abuse cases in Spain have increased by 140% since 2011 [9].

According to official data, among the reasons for alcohol consumption, the one associated with socialization predominates, although this cause decreases in adult consumers, who also use alcohol because of its effects after its ingestion such as euphoria, excitement, loquacity, or disinhibition, among others [2]. 

One of the main mechanisms used by premium alcohol brands to influence consumer behavior is advertising [10,11,12]. A total of 28% of Spanish people pay special attention to alcoholic beverage brands, which is behind the categories of cosmetics and body care (35%) [13].

However, despite the legal limitations imposed on advertising for alcoholic beverages, especially in audiovisual media, the rates obtained by Spain in terms of adequate protection are very low: only 18 out of a maximum of 60 [14]. Thus, according to the Spanish Observatory of Drugs and Addictions, 59.5% of the Spanish population considers that advertising prohibition should be a fundamental measure to avoid higher consumption rates [2]. 

### 1.2. Online Gambling as an Addictive Behavior

Nowadays, gambling has become an important social demand that influences citizens [15], being the trigger of addiction problems such as ludopathy and domestic economic problems.

The Diagnostic and Statistical Manual of Mental Disorders (DSM-5) catalogs as “highly addictive” the pathological gambling on disorders that are not directly related to substance use [16]. Volberg et al. [17] and Armstrong and Carroll [18] explained that the fact that online gambling and sports betting had become established in the lives of many young people as an increasingly popular and normalized pastime, leading them to become addicted, was closely related to the coverage that many media and even sports teams have given to these platforms.

Additionally, sports betting is for many people a way to increase their enjoyment while watching sports events, and it is increasingly common to find exposure to the opportunities offered by gambling through media advertising, which has led to concerns about the ability of such advertising to regulate gambling behavior [19]. These advertisements emphasize sports gambling’s positive factors, showing a high level of marketing that increases the desire to gamble among players who have a problem or addiction. 

Unlike other games of chance, these bets are based on the fact that the sport—and its results—is independent of the randomness of such games. However, the gambler interacts with a broader range of elements of psychosocial importance including sporting randomness, sporting team identity, television visualization of sports (i.e., media psychology, sports-related behaviors) such as community visualization, junk food consumption, and alcohol consumption as well as emotionally charged situations [20].

According to Bet365, considered the world’s largest digital betting company, up to 80% of their sports betting revenue comes from live betting [21]. As a result, several European jurisdictions have banned or reduced live betting due to its proven addiction [22,23]. Furthermore, countries such as the United Kingdom have banned the use of credit cards exclusively for online platforms, precisely to try to reduce the damage, both economic and personal, caused by live betting [24].

The social construction of the “normal bettor” by mass media and gambling normalization could lead to an increase in gambling-related harm [25,26,27]. In many countries and jurisdictions, gambling is considered entertainment, characterized by betting/wagering mechanics and monetization features. However, according to the World Health Organization (WHO), gambling disorder is categorized as a disorder due to addictive behaviors associated with distress or impairment in the International Classification of Diseases (ICD-11) in 2019. Although bets/wagers cannot be considered drugs, excessive gambling promotion, where advertising plays an important role, can lead to an addictive consumption.

### 1.3. Online Betting in Spain

With the legalization of most of the games of chance in Spain in 1977, the adverse effects and addictive behaviors have been constant. According to Labrador and Vallejo-Achón [28], 91% of the adult population admits to having gambled, especially in recent years, where young people’s frequency of betting has increased. On the other hand, concerning the traditional pre-game bets, they have increased the live bets, that is, those made when the event has already started. These bets have become the most popular among gamblers and represent up to 59% of the money circulating in sports betting currently made in Spain [20].

For the online gaming market in Spain, the gross gaming revenue (GGR), the amount dedicated to participation in the game (deducting the bonuses and prizes paid by the operator to the participants) was 699 million euros in 2018, which represents an increase of 25.5% over the previous year [29]. The Spanish bet almost 7000 million euros in 2018 compared to the 2010 million euros in 2013. The betting market accounts for 52.2% of the total gaming market, and of that percentage, 41.59% corresponds to conventional sports betting and 55.9% to live sports betting. The preference for this last modality is reflected in the tendency shown by the figures played by the Spanish in the last years: in conventional sports bets, it has gone from 2819 million euros in 2014 to 2014 in 2018, however, live sports bets have grown from 39,700,000 euros in 2014, to 4,753,474,076 euros in 2018 [29]. 

On the other hand, investment in marketing represented 328 million euros in 2018, which is an increase of 48% compared to 2017. Likewise, advertising accounts for 51.16% of this market, with an increase in the advertising expenditure notably growing every year, going from 68 million euros in 2013 to 168 million euros in 2018 [29]. Some of the most widely used advertising formats for sports betting operators are adverts embedded in match commentary and commercial breaks on radio and television [30].

### 1.4. Effects of Alcohol Portrayal in the Media: State-of-the-Art

The media and its contents significantly impact our daily lives, especially on vulnerable populations such as children and adolescents, who are in a vital stage of change and shape their own identity. Given the potential risk of exposure to problematic content such as drug consumption or dangerous attitudes regarding health, there have been many scientific community contributions. However, most of them deal with alcohol in audiovisual media, especially in soap operas and movies.

Pendleton, Smith, and Roberts [31] analyzed 50 British television programs in 1988, showing that three-quarters of the programming included visual or verbal references to alcoholic beverages, which on average meant one reference to this type of product every 6.5 min. Coyne and Ahmed [32] agreed with this research in an analysis of the presence of smoking and alcoholic beverages in soap operas on British television, demonstrating that more than 90% of the episodes analyzed showed the non-moderate consumption of alcohol without consequences. These two studies claim that displaying these behaviors naturally, especially by young prescribers, tends to normalize conduct without understanding the adverse health effects that indiscriminate consumption can generate.

In the same spirit, Mathios et al. [33] analyzed the fictional programming of American prime time television, showing that alcoholic beverages were the most common form of consumption of food and beverages, even in teenage characters. However, they went further, specifying that the characters who appeared to be more successful tended to be more active consumers, which they warn may encourage the audience’s consumption. Russell, Russell, and Grube [34] agreed with this and analyzed, on one hand, the messages and appearances of alcoholic beverages in the American series The O.C., and on the other hand, the perception of the absence of messages and the product placement of alcoholic beverages and its effect on attitudes, beliefs, and behaviors. This research showed that despite repeated consumption in the television series, viewers were more receptive to the positive messages of the moderation of consumption. 

It is necessary to point out that the audience’s attitudes and behaviors can be a mediator of the perception of alcohol consumption in the media, as shown by Mayrhofer and Matthes [35] from an experimental study. This research concluded that those who consumed few alcoholic beverages perceived themselves as more similar to characters in a fictional series who drank little, demonstrating that the perceived similarity and the audience’s alcohol consumption—at the individual scale—are intermediaries of their level of affectation.

However, van Hoof, from Jong and Gossett [36], in an experimental study with adolescents, showed that exposure to alcohol consumption in fictional programming (such as soap operas) revealed a negative attitude of the participants toward its consumption, although in the case of advertising for alcoholic beverages, the opposite happens: it encourages consumption and increases attitudes toward unhealthy consumption behaviors.

### 1.5. Social Responsibility of Advertisers and Media and the Danger of Self-Regulation

The relationship between the media and society has always been problematic in two areas. First, the media control and influence society through priming, agenda-setting, framing, and advertising, according to their commercial and political interests, while, second, the enormous changes and dynamics of information technologies make media standards and regulations obsolete very quickly [37,38].

Thus, the media, like other types of organizations, are not exempt from committing faults such as fraud, theft, misinformation, broken promises, and broken law regulations [39], which generally results in a lack of social trust toward media corporations, even though many of them invest in public relations strategies [40]. This distrust is increasing in the current times as media companies have been growing gradually, and even their ownership has been concentrated in giant international empires whose financial and ownership structures are opaque in the light of society [41]

The social responsibility of the media and advertisers can be interpreted both in terms of “responsibility”, relating to their commitment to society, and “responsiveness”, relating to how the media and the advertiser listen to and take into account—ethically—their audience [42]. Therefore, responsibility has to do with compliance with appropriate conduct, national and international legislation, ethical standards of procedures, and accountability [43]. 

Terms such as “accountability”, “responsibility”, and “responsiveness” of the media have been gradually included in the political and legislative agenda of many European countries seeking to improve the formal and informal procedures used by broadcasters, regulators, or self-regulated bodies to take into account the wishes and needs of the audience, primarily because the media can have adverse social effects and, consequently, must bear the social costs they generate [44]. Of course, these changes occur at different rates in various European countries, although the trend of regulation and accountability is the most prevalent.

Despite the many benefits that McQuail [45] found in professional responsibility and media self-regulation, mainly because it guarantees a greater degree of freedom, under the current reality, the interests of media owners, shareholders, and advertisers are imposed, so it is common to observe illegal and unethical practices in media content such as advertising for high alcohol and betting at restricted times. This raised the initial research question of the study (RQ0): Does the radio media in Spain comply with the legislation and legal restrictions on the broadcasting of advertising for high alcoholic beverages and sports betting? 

In fact, according to the results of the study by [46], carried out in 14 European countries on 1762 journalists, the effectiveness of self-regulation practices can be questioned—except for Finland and Switzerland—with the internal rules of the journalistic enterprise being the most important, followed by legal regulations. Although these results are striking, in the same study, the information professionals surveyed assured that more significant state intervention was not desirable, so there is a need to establish self-regulation mechanisms that include sanctions, a type of mixed regulatory regime called “co-regulation” [47].

Unlike the United States, where the media are more sensitive to media accountability, in Europe, self-regulation in advertising is traditionally important [48,49], allowing the European Commission to regulate these policies freely. Other studies support that some European countries such as Germany, Finland, and the Netherlands follow a correct model of accountability [50,51] within a consensual political system that has generated a strong tradition of public service media.

For their side, the advertisers are the first ones responsible before the audience of all their commercial communication, understanding that they are the ones who define and approve the contents of the ads, the message, their codes, the choice of the radio station, and the time of the broadcast [52].

The development of malpractices of social responsibility by advertisers in the media results in medium- and long-term damage to the perception, image, and reputation that the stakeholders have of their associated brands [53,54], so the interested parties usually demand responsible advertising following the social interests and needs. 

However, few precedents have linked alcohol and gambling advertising with a special focus on minors and the protected time slot despite the problems above-mentioned. 

Thomas et al. [55] showed that 67.6% of young people recalled seeing gambling advertising in the early evening before 8:30 pm; meanwhile, Jones et al. [25] highlighted that children and young people were also exposed to gambling and alcohol advertising because broadcast media offered a loophole to avoid the post-watershed guidelines. On the other hand, [20] found that betting and risk behaviors such as consuming alcohol were associated and also that betting advertising aligns with drinking alcohol and significantly associates emotionally charged sporting situations such as watching live games or celebrating goals with alcohol [26,56]. 

In the previous studies analyzed, those of an experimental nature predominate, and they have tended to focus on television media. However, no studies have been found that have linked the advertising of high-grade alcoholic beverages and betting on the radio. In this sense, the main objective of this research was to compare the presence of advertising on the radio of two products that represent harmful behaviors for health (gambling and high alcoholic beverages advertising) and to verify if the media and advertisers complied with the schedule of the protection of minors regarding the limitation of broadcasting this type of advertising.

### 1.6. Regulation of High Alcohol Beverages and Betting Radio Advertising

In Spain, there is no specific legislation regulating the advertising of high-grade drinks or online betting. In this regard, Article 5, Section 5 of Law 34/1988, of November 11, on Advertising, prohibits the advertising of beverages with an alcoholic graduation higher than 20º on television. This same limitation is also found in Law 7/2010, of March 31, on General of Audiovisual Communication in its Article 18, paragraph 3c. 

However, Section 3 of the aforementioned Article 18, which refers to commercial communications prohibited in any form, generally forbids commercial communications that encourage behavior that is harmful to health. This limitation affects both the consumption of high-grade drinks and betting, which are promoted by advertising these types of products. Likewise, the limitation to broadcast advertising of high-grade beverages regarding their consumption represents a potential health risk. Therefore, it is extended and applicable to the radio and not only to television, as explicitly stated in the paragraph above-mentioned. Previous administrative sanctions [57] indicate that the general limitations on both radio and television must be understood as applicable to alcoholic beverage advertising, regardless of its alcohol content. 

Finally, concerning the protection of minors, Article 7 of the aforementioned Law 7/2010, of 31 March, on General of Audiovisual Communication, sets a time slot from 06:00 to 21:59, in which no content may be broadcast openly that could harm the physical, mental, or moral development of children. In this sense, although the recipients of spirits drinks and betting advertising in full-service radio stations are adults, this law seeks to protect minors to prevent them from listening to advertising that could pose a risk in the formation of false perceptions about the reality of the consumption of these types of products. These legal parameters and the general objective of the investigation led to the following research questions (RQ):

- *RQ 1:* What is the presence of spirits drinks and betting advertising on news/talk radio stations?
- *RQ 2:* What is the presence of spirits drinks and betting advertising by format on news/talk radio stations?
- *RQ 3:* Do news/talk radio stations respect the minor protection time slot in the case of broadcast spirits drinks and betting advertising?
- *RQ 4:* Is there any relation between news/talk radio stations and minor protection time slots by different advertising formats?

## 2. Materials and Methods

The methodology chosen to develop this work followed a quantitative approach based on content analysis, enabling the objective and systematic content description [58] of all radio spots and mentions broadcast throughout 2017 in Spain. We chose the news/talk radio station format since the program content is based on news and current affairs and listeners pay more attention to radio information than music. The selection of the stations followed two criteria: national coverage and Spanish-language broadcasting. According to data from the Estudio General de Medios [Media General Study] (EGM) [59], the stations with the highest audience levels were Cadena Ser, Cadena Cope, and Onda Cero, with a total of 9,000,000 daily listeners.

Another methodological decision was to differentiate the analysis according to the two most frequent advertising formats in Spanish radio, but with significant differences regarding their broadcast and characteristics: (1) radio spots were defined as a pre-recorded message between 20 and 30 s long broadcast during a commercial break and separated from the programming so listeners can distinguish between advertising and editorial contents; and (2) radio mentions were advertisements read live by the presenters, team, or co-workers of programs embedded within the programming, usually with an absence of content separation lines or sonic triggers that would warn listeners of its commercial nature. Thus, this advertising format involves some ethical concerns. The final corpus was comprised of a total of 2848 radio messages distributed as follows: 266 radio spots and 2582 radio mentions.

The data analyzed were obtained from Arce Media’s database (joined since 2007 to Nielsen’s database), a company dedicated to collecting and analyzing advertising activity in conventional media.

- News/talks Radio Stations: (1) Cadena Ser; (2) Cadena Cope; (3) Onda Cero.
- Type of product: (1) High alcohol beverages: brandy, gin, rum, vodka, whisky, other liquors; (2) Betting: online betting, sports betting.
- Advertising Format: (1) Spots: (2) Mentions.
- Special Protection Time Slot: (1) Protected Time Slot (from 6:00 to 21:59); (2) Non-protected Time Slot (from 22:00 to 5:59).

The complete analysis and coding process were carried out by two trained coders, codified according to the variables and their attributes. The variables studied were mostly of a structural nature; therefore, their codification did not require an intersubjective interpretation. In any case, the inter-codifier reliability was measured using Cohen’s Kappa [60], which raised a variation between 0.968 and 1, calculated with SPSS’17 (IBM, Armonk, NY, USA). Aside from the structural variables Radio Station, Type of Product, and Special Protection Time Slot (all of them *k* = 1), the variable Advertising Format obtained a value of *k* = 0.968. To solve this detected divergence, a third work session was made. The few discrepancies were based on codification errors, and not on interpretation ones. After evaluating the cases, the final coding was decided by the two coders. The results shown below were based on a value k = 1 for all variables. Furthermore, any crossed data of the coded variables were submitted to relevant statistical significance tests using nonparametric χ^2^ analysis. 

## 3. Results 

The analysis results linked the variables that articulated the research questions from the set of all radio spots and radio mentions broadcast during 2017 on high-grade alcoholic beverages (544) and online and sports betting (2304), with a total of 2848 advertisements analyzed. 

Regarding RQ1, the presence of spirits drinks and betting advertising on news/talk radio stations, the total corpus understudy was distributed in a percentage of 19.1% and 80.9%, respectively. The news/talk radio stations responsible for broadcast advertising presented a very homogeneous proportion across the whole of the corpus analyzed. Cadena Cope, Cadena Ser, and Onda Cero accounted for 33.3%, 34.6%, and 32.1%, respectively, of the advertising broadcasts studied. Table 1 shows that advertising referring to bets presented a frequency four times higher than that of alcoholic beverages (80.9% versus 19.1%).

**Figure 1:**
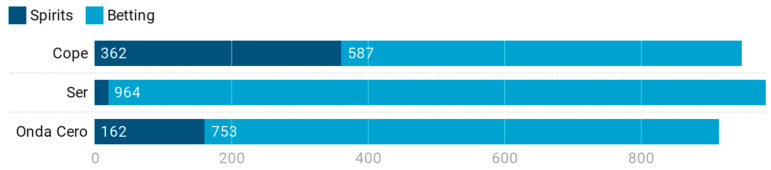
Spirits and betting advertising by news/talk radio stations in Spain.

This distribution—always favorable to betting advertising—had more relevance in Cadena Ser. At this radio station, ads and mentions referring to spirits drinks barely reached 2%, and the rest, 98%, was betting advertising, so much so that Cadena Ser accumulated 41.8% of all betting advertising broadcast by the three news/talk radio stations that were the object of this study. However, Cadena Cope broadcasted more spirits drinks advertising with 66.5% of the total. Finally, Onda Cero broadcasted 82.3% of betting advertising and accumulated 29.8% of total advertising for high alcoholic beverages in the entire sample studied (Figure 1).

**Figure 2:**
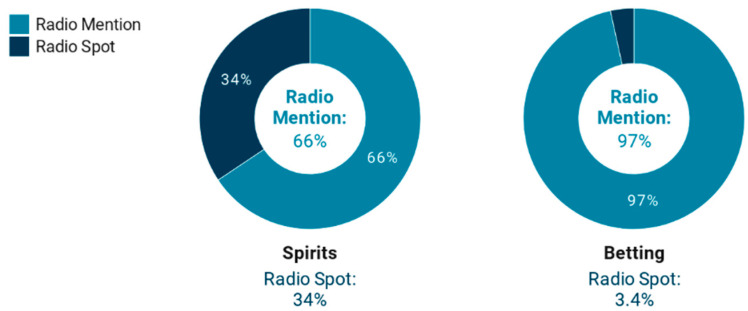
Spirits and betting advertising by advertising format on news/talk radio stations.

RQ2 in this research aimed to understand the presence of spirits drinks and betting advertising by format on news/talk radio stations. The results showed some very significant particularities. As previously mentioned, 90.7% of the advertising analyzed corresponded to the radio mention type message (2582), while 9.3% (266) was advertising by format on radio spots. As shown in Table 2, the latter accounted for 34.4% of advertising for high alcohol beverages, while radio mentions accounted for the remaining 65.6%. Therefore, radio spots obtained a proportion of spirits drinks advertising that was much higher than the relative weight they represented in the corpus analyzed. A total of 70.3% of all advertising for this type of alcoholic beverage was advertised through radio spots (Table 2). In contrast, 86.2% of all betting advertising broadcasts in the reference period were concentrated in radio mentions (Figure 2).

RQ3 aimed to explore the presence of this type of advertising broadcasts and the schedules to protect minors established in Spanish legislation. Table 3 provides an answer to this question by offering an initial clarifying key figure: 88.6% of the radio advertising content analyzed referring to spirits drinks and betting was broadcast during protected hours (06:00–21:59) (Table 3). These data show the possible exposure of minors to a type of advertising that encourages consumption typologies that are potentially dangerous for their health.

**Figure 3:**
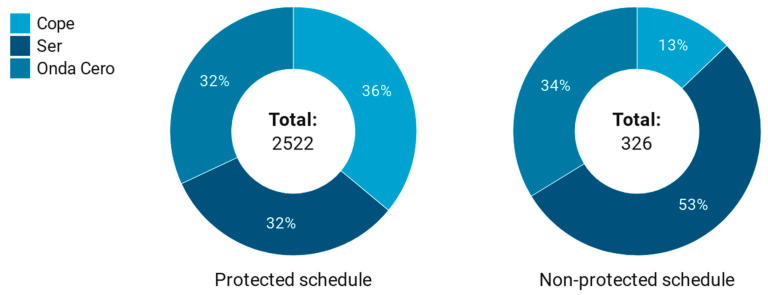
News/talk radio station by minor protection time slot of spirits and betting advertising in Spain.

Disaggregating by news/talk radio stations, Cadena Cope was the station that most frequently infringed the protected schedule of minors, to the point that 95.6% of the spirits drinks and betting advertisements broadcast in 2017 were concentrated in the 06:00–21:59 range. This percentage reached 88% in the case of Onda Cero and 82.3% in Cadena Ser. Without a doubt, all three had remarkably high percentages. However, if we compare the broadcasting frequencies among the three news/talk radio stations analyzed, the presence of spirits drinks and betting advertising was very similar among them, although once again, Cadena Cope stood out with the highest percentage of this type of advertising broadcast during protected hours, precisely 36% of the total, followed by Cadena Ser and Onda Cero with 32.1% and 31.9%, respectively (Figure 3).

RQ4 sought to deepen the possible existence of a relationship between news/talk radio stations and minor protection time slots by different advertising formats. For this purpose, we carried out a residual analysis. This type of analysis verifies whether there is an especially relevant relationship of attraction or rejection between two variables that have passed a χ^2^ test of statistical significance. The aim was to identify the existence of anomalous cases that had a “pattern of relationships significantly different from that of the majority of cases observed” (Sánchez Carrión 1999, 341) [61].

Table 4 highlights the particular intensity of the advertising broadcasts of spirits drinks and betting radio spots on Cadena Cope in the protected time zone. A statistical residue of 4.8 implies a particularly significant correlation force that is much more powerful than that reflected in the other two stations under study. These data are entirely consistent with the frequency distribution percentages. As shown in Table 4, 98.1% of all the commercial breaks of this type broadcast by Cadena Cope were concentrated in protected hours, although Onda Cero presented a higher percentage compared with the other stations (47.3%). 

The analysis referring to the radio mention format is shown in Table 5, highlighting the particular relevance of the Cadena Cope broadcasts in the protected time-slot. A statistical residue of 7.2 indicates an extraordinarily strong association between spirits drinks and betting radio mentions on this station, and 95.3% of the broadcasts in this format took place during the protected time-slot. Likewise, Cadena Cope accumulated 35.1% of all mentions of this type in the reference year (2017), which was higher than Cadena Ser (34.5%) and Onda Cero (30.4%).

These data show that the main radio stations in Spain broadcast advertising for high alcoholic beverages and sports betting, mainly through the format of mentions, and did not respect the legal limitations established by the schedule for the protection of minors.

## 4. Discussion

The research conducted compared the behavior of advertisers of products whose consumption may represent a risk to health and the radio media regarding respecting the schedule of the protection of minors that is subject to regulation. This restriction aims to prevent children and young people from being reached by advertisements that normalize high alcohol content consumption and betting. Alcohol is a drug considered legal, whose abuse leads to addiction, while gambling is a behavior considered addictive, recently cataloged by the WHO.

The results of the analysis showed a media reality that deserves deep reflection. First, the high presence of betting advertising compared to alcohol, which was eight out of 10. It is true that traditionally and for a long time, high alcoholic beverages have suffered from a more significant restriction on sales and advertising regulated by law and that betting has been increasing its presence in the media over time, and advertisers have also increased their income, since to date, there are no specific laws regulating this type of commercial communication in Spain or in Europe. 

Given the consequences that most countries show after abusive behavior that leads to addiction in the adult population and the youngest, many countries are already developing laws that limit this type of advertising, especially on television. In Spain, the approval of a law on regulating the communication of sports bets in audiovisual media (i.e., radio and television) is foreseen for the end of 2020. This project poses severe restrictions both concerning the content and broadcast of such advertisements.

On the other hand, the three analyzed stations broadcast a very similar percentage of spirits drinks and betting advertising. However, it is worth noting that nine out of every 10 advertisements in these two product categories broadcast in 2017 on Spanish radio were mentions (i.e., advertising integrated into the live program and carried out by the radio hosts or collaborators themselves). The importance of these data lies in the fact that the listener is prevented from avoiding the advertisement if they do not want to listen to it. In this sense, radio mentions are the predominant format in betting advertising and implies important ethical considerations for the journalists who collaborate in its realization [62] as well as the questionable social responsibility of the medium toward its audience [43]. 

Regarding the article’s central variable that analyzed compliance with the legislation regarding child protection schedules, the results showed almost nine out of 10 spirits drinks and betting ads analyzed were broadcast during protected time slots. It is essential to point out the high impact that sports broadcasts had on the study and the biases associated with radio broadcasts. For some years now, the enormous investments in television rights made by the big operators have forced an extension of the days and time slots in which sports events are broadcast, especially soccer. All this is to avoid overlaps in the transmissions, maximizing the return of these investments via pay-per-view and, by extension, guaranteeing the clubs’ income. 

This reality has forced radio stations to extend their sports broadcasts over entire days, especially on weekends. Both national and international competitions are affected by this extensive sports broadcasting model, which has a significant impact not only on the type of advertising that is broadcast, but also on its format, with the mentions being the most benefited format. It is a much more dynamic format than the traditional commercials, and it fits much better in radio productions that can reach up to 16 h of uninterrupted broadcasting. 

Although the objective of this research was not to analyze the effects that exposure to this type of message had on the audience, but to show that the self-regulation of the media in terms of compliance with their legal and ethical obligations could generate continuous infringements that can affect the development and health of particularly vulnerable populations such as children and adolescents, it is noted, in line with the research findings of van Hoof, de Jong, and Gossett [36], that alcohol advertising encourages audience attitudes toward non-moderate consumption, so its regulation and enforcement should be a public health issue.

### 4.1. Implications

Previous studies have questioned the effectiveness of advertising self-regulation systems in general and in Spain [63], demanding greater restrictions in the vast majority of media to prevent the exposure of young people to this type of advertising [55]. Even journalists themselves have called for more effective co-regulation [46]. However, this study joins other previous ones that have shown that radio journalists in Spain do not respect one of the main ethical principles that regulate their profession’s practice, which is the one referring to their participation in advertising.

In any case, the public administration should firmly consider a complete prohibition of spirits drinks advertising as demanded by the vast majority of Spanish people [2] and advertising of these types of products should have similar limitations to those of tobacco. 

Above all, there is an urgent need for common regulations in the European environment and internationally, given that communications are now global and any radio or television broadcast can be accessed and received in any country in the world at the same time that it is being broadcast. There is no point in having severe limitations in one country and lax ones when Internet borders have disappeared for audiovisual communication. Moreover, if we focus on sports broadcasting, the audiences for certain matches reach millions worldwide. 

### 4.2. Limitations and Future Research

This study’s main limitation is that it focused on a type of radio station with an adult audience and whose programming was based on information, so future work should compare results with music stations whose audience profile is much younger. 

Another limitation is that the analysis was not disaggregated by product type, and it could be of interest to know which spirits drinks and betting type predominate at this type of station. Additionally, given the growing investment in sports betting [20], future work should focus on this type of advertising, especially on the radio and television during the transmission of sports programs.

The main focus of this paper was on the regulation of child protection schedules. Future studies may also complete the analysis with other legislative restrictions on advertising these types of products. Likewise, it would be interesting to compare the data obtained in this work with analyses in other media such as television or magazines where the target can also be segmented. 

As our research focused on a national legislative rule that is still a transposition of a European directive, it would also be interesting to analyze the study object from its application in other European countries.

## 5. Conclusions

The advertising of alcoholic beverages, and especially sports betting, takes on a role that has never been known before, entering into serious contradiction with the current legislation. It is precisely the need to finance increasingly expensive radio productions, for more and more hours, which explains the broadcasting of this type of advertising in unauthorized time slots, especially on weekends. However, the media’s necessary financing cannot be achieved by skipping professional ethical codes and behaving irresponsibly toward the audience, which in this research was predominantly adult. Nevertheless, even minors and young people can be impacted by undesirable advertising, as previous studies have shown [25,55]. 

This exposure to advertising of these types of products can generate false expectations and incorrectly educate them given the associations with which advertising presents the consumption of spirits drinks and betting.

## Figures and Tables

**Table 1:** Spirits and betting advertising by News/talk radio stations in Spain.

		Product		Total
		Spirits	Betting	
**Cope**	*n*	362	587	949
	% horizontal	38.1	61.9	100.0
	% vertical	66.5	25.5	33.3
**Ser**	*n*	20	964	984
	% horizontal	2.0	98.0	100.0
	% vertical	3.7	41.8	34.6
**Onda Cero**	*n*	162	753	915
	% horizontal	17.7	82.3	100.0
	% vertical	29.8	32.7	32.1
**Total**	*n*	544	2304	2848
	% horizontal	19.1	80.9	100.0
	% vertical	100.0	100.0	100.0

Note: χ^2^ (2, *n* = 2848) = 409,412, *p* < 0.001.

**Table 2:** Spirits and betting advertising by advertising format on news/talk radio stations.

		Product		Total
		Spirits	Betting	
**Radio Spot**	*n*	187	79	266
	% horizontal	70.3	29.7	100.0
	% vertical	34.4	3.4	9.3
**Radio Mention**	*n*	357	2225	2582
	% horizontal	13.8	86.2	100.0
	% vertical	65.6	96.6	90.7
**Total**	*n*	544	2304	2848
	% horizontal	19.1	80.9	100.0
	% vertical	100.0	100.0	100.0

Note: χ^2^ (1, *n* = 2848) = 497,734, *p* < 0.001.

**Table 3:** News/talk radio stations by minor protection time slot of spirits and betting advertising in Spain.

		Time Protection		Total
		Protected Schedule (06:00–21:59)	Non-Protected Schedule (22:00–05:59)	
**Cope**	*n*	907	42	949
	% horizontal	95.6	4.4	100.0
	% vertical	36.0	12.9	33.3
**Ser**	*n*	810	174	984
	% horizontal	82.3	17.7	100.0
	% vertical	32.1	53.4	34.6
**Onda Cero**	*n*	805	110	915
	% horizontal	88.0	12.0	100.0
	% vertical	31.9	33.7	32.1
**Total**	*n*	2522	326	2848
	% horizontal	88.6	11.4	100.0
	% vertical	100.0	100.0	100.0

Note: χ^2^ (2, *n* = 2848) = 84,203, *p* < 0.001.

**Table 4:** News/talk radio stations by minor protection time slot of spirits and betting radio spots in Spain. Residuals.

		Time Protection		Total
		Protected Schedule (06:00–21:59)	Non-Protected Schedule (22:00–05:59)	
**Cope**	*n*	101	2	103
	% horizontal	98.1	1.9	100.0
	% vertical	44.7	5.0	38.7
	Residuals	4.8	−4.8	
**Ser**	*n*	18	8	26
	% horizontal	69.2	30.8	100.0
	% vertical	8.0	20.0	9.8
	Residuals	−2.4	2.4	
**Onda Cero**	*n*	107	30	137
	% horizontal	78.1	21.9	100.0
	% vertical	47.3	75.0	51.5
	Residuals	−3.2	3.2	
**Total**	*n*	226	40	266
	% horizontal	85.0	15.0	100.0
	% vertical	100.0	100.0	100.0

Note: χ^2^ (2, *n* = 2848) = 23,909, *p* < 0.001.

**Table 5:** News/talk radio stations by minor protection time slot of spirits and betting radio mentions in Spain. Residuals.

		Time Protection		Total
		Protected Schedule (06:00–21:59)	Non-Protected Schedule (22:00–05:59)	
**Cope**	*n*	806	40	846
	% horizontal	95.3	4.7	100.0
	% vertical	35.1	14.0	32.8
	Residuals	7.2	−7.2	
**Ser**	*n*	792	166	958
	% horizontal	82.7	17.3	100.0
	% vertical	34.5	58.0	37.1
	Residuals	−7.8	7.8	
**Onda Cero**	*n*	698	80	778
	% horizontal	89.7	10.3	100.0
	% vertical	30.4	28.0	30.1
	Residuals	0.8	−0.8	
**Total**	*n*	2296	286	2582
	% horizontal	88.9	11.1	100.0
	% vertical	100.0	100.0	100.0

Note: χ^2^ (2, *n* = 2848) = 73,121, *p* < 0.001.

## References

[1] (2018). WHO (World Health Organization) Global Status Report on Alcohol and Health 2018. https://apps.who.int/iris/bitstream/handle/10665/274603/9789241565639-eng.pdf?ua=1.

[2] Ministerio de Sanidad, Consumo y Bienestar Social. Observatorio Español de Drogas y Adicciones (OEDA) (2019). Encuesta Sobre Alcohol y Drogas en España. Alcohol, Tabaco y Drogas Ilegales en España. http://www.pnsd.mscbs.gob.es/profesionales/sistemasInformacion/informesEstadisticas/pdf/2019OEDA-INFORME.pdf.

[3] Kantar Media TGI Survey Data 2019. https://www.kantarmedia.com/global/our-solutions/consumer-and-audience-targeting/tgi-survey-data.

[4] INE (Instituto Nacional de Estadística) (2018). Encuesta Nacional de Salud. Determinantes de la Salud.

[5] Euromonitor International (July 2019) Spirits in Spain. Country Report..

[6] FEBE (Federación Española de Bebidas Espirituosas) (2020). El Sector en Cifras. https://www.espirituosos.es/El-sector-en-cifras/Datos-de-interes/#.

[7] Infoadex E. Estudio InfoAdex de la Inversión Publicitaria en España 2019. https://www.infoadex.es/home/estudio-infoadex-de-la-inversion-publicitaria-en-espana-2019/.

[8] INE (Instituto Nacional de Estadística) (2020). Encuesta de Morbilidad Hospitalaria 2018.

[9] Ministerio Sanidad, Consumo y Bienestar Social (2020). Base de Datos Clínicos de Atención Primaria.

[10] Atkin C.K., Neuendorf K., McDermott S. (1983). The role of alcohol advertising in excessive and hazardous drinking. J. Drug Educ..

[11] Saffer H. (1996). Studying the effects of alcohol advertising on consumption. Alcohol Health Res. World.

[12] Houben K., Schoenmakers T.M., Wiers R.W. (2010). I didn’t feel like drinking but I don’t know why: The effects of evaluative conditioning on alcohol-related attitudes, craving and behavior. Addict. Behav..

[13] Statista (2019). Global Consumer Survey. https://www.statista.com/getting-started/more-statista-services-global-consumer-survey.

[14] European Policy Information Center (2019). The Nanny State Index. http://nannystateindex.org/alcohol-2019/#.

[15] Seelig M.Y., Seelig J.H. (1998). Place your bets on gambling, government and society. Can. Public Policy.

[16] Buil P., Solé M.J., García P. (2015). Online Gambling Advertising Regulations in Spain. A Study on the Protection of Minors. Adicciones.

[17] Volberg R.A., Gupta R., Griffiths M.D., Olason D.T., Delfabbro P. (2010). An international perspective on youth gambling prevalence studies. Int. J. Adolesc. Med. Health.

[18] Armstrong A.R., Carroll M. (2017). Sports Betting in Australia.

[19] Lole L., Russell A., Li E., Thorne H., Greer N., Hing N. (2019). Interest in inducements: A psychophysiological study on sports betting advertising. Int. J. Psychophysiol..

[20] López-Gonzalez H., Griffiths M.D., Estévez A. (2020). In-Play Betting, Sport Broadcasts, and Gambling Severity: A Survey Study of Spanish Sports Bettors on the Risks of Betting on Sport While Watching It. Commun. Sport.

[21] Killick E.A., Griffiths M.D. (2019). In-Play Sports Betting: A Scoping Study. Int. J. Ment. Health Addict..

[22] Oliveri N. (2012). Online gambling. Double standards in French discourse on addiction. Hermes.

[23] Chóliz M., Lamas J. (2017). “Place your bets, children!” The frequency of gambling among minors and their relationship with gambling addiction indicators. Rev. Española Drogodepend..

[24] Hing N., Russell A.M.T., Li E., Vitartas P. (2018). Does the uptake of wagering inducements predict impulse betting on sport?. J. Behav. Addict..

[25] Jones C., Pinder R., Robinson G. (2020). Gambling sponsorship and advertising in British football: A critical account. Sport Ethics Philos..

[26] Lopez-Gonzalez H., Estévez A., Griffiths M.D. (2018). Controlling the illusion of control: A grounded theory of sports betting advertising in the UK. Int. Gambl. Stud..

[27] Lopez-Gonzalez H., Estévez A., Griffiths M.D. (2019). Can positive social perception and reduced stigma be a problem in sports betting? A qualitative focus group study with Spanish sports bettors undergoing treatment for gambling disorder. J. Gambl. Stud..

[28] Labrador F.J., Vallejo-Achón M. (2019). Prevalence and characteristics of sports betting by young Madrid students. J. Gambl. Stud..

[29] DGOJ (Dirección General de Ordenación del Juego) (2019). Mercado de Juego Online Estatal, 2018. https://www.ordenacionjuego.es/es/mercado-juego-online-estatal.

[30] Lopez-Gonzalez H., Guerrero-Solé F., Griffiths M.D. (2018). A content analysis of how ‘normal’ sports betting behaviour is represented in gambling advertising. Addict. Res. Theory.

[31] Pendleton L., Smith C., Roberts J.L. (1991). Drinking on television: A content analysis of recent alcohol portrayal. Br. J. Addict..

[32] Coyne S.M., Ahmed T. (2009). Fancy a pint? Alcohol use and smoking in soap operas. Addict. Res..

[33] Mathios A., Avery R., Bisogni C., Shanahan J. (1998). Alcohol Portrayal on Prime-Time Television: Manifest and Latent Messages. J. Stud. Alcohol.

[34] Russell C.A., Russell D.W., Grube J.W. (2009). Nature and Impact of Alcohol Messages in Youth-Oriented Television Series. J. Advert..

[35] Mayrhofer M., Matthes J. (2020). Observational learning of the televised consequences of drinking alcohol: Exploring the role of perceived similarity. Nord. Stud. Alcohol Drugs.

[36] van Hoof J.J., de Jong M.D.T., Fennis B.M., Gosselt J.F. (2009). There’s alcohol in my soap: Portrayal and effects of alcohol use in a popular television series. Health Educ. Res..

[37] Romero-Rodríguez L.M., de-Casas-Moreno P., Torres-Toukoumidis A. (2016). Dimensions and Indicators of the Information Quality in Digital Media. Comunicar.

[38] Romero-Rodríguez L.M., Civila S., Aguaded I. (2020). Otherness as a form of intersubjective social exclusion: Conceptual discussion from the current communicative scenario. J. Inf. Commun. Ethics Soc..

[39] Green S.P. (2007). Lying, Cheating, and Stealing: A Moral Theory of White-Collar Crime.

[40] Gulyás Á. (2011). Demons into angels? Corporate social responsibility and media organisations. Crit. Surv..

[41] Wilenius M., Malmelin N. (2009). Towards sustainably managed media organizations: Reflections on the future of responsible business in media industry. Bus. Strategy Ser..

[42] Hermans L., Schaap G., Bardoel J. (2014). Re-establishing the relationship with the public: Regional journalism and citizens’ involvement in the news. J. Stud..

[43] Bardoel J., D’Haenens L. (2004). Media responsibility and accountability: New conceptualizations and practices. Comunications.

[44] Baldi P., Baldi P., Hasebrink U. (2007). Media accountability in Europe: A fragmented picture. Broadcasters and Citizens in Europe: Trends in Media Accountability and Viewer Participation.

[45] McQuail D. (2005). McQuail’s Mass Communication Theory.

[46] Fengler S., Eberwein T., Alsius S., Baisnée O., Bichler K., Dobek-Ostrowska B., Evers H., Glowacki M., Groenhart H., Harro-Loit H. (2015). How effective is media self-regulation? Results from a comparative survey of European journalists. Eur. J. Commun..

[47] Prosser T. (2008). Self-regulation, co-regulation and the audiovisual media services directive. J. Consum. Policy.

[48] Harrison J., Woods L.M. (2001). Defining European public broadcasting. Eur. J. Commun..

[49] Potschka C. (2012). Towards a Market in Broadcasting: Communications Policy in the UK and Germany.

[50] De Haan Y., Bardoel J. (2011). From trust to accountability: Negotiating media performance in the Netherlands, 1987–2007. Eur. J. Commun..

[51] Nielsen R.K., Linnebank G. (2011). Public Support for the Media: A Six-Country Overview of Direct and Indirect Subsidies.

[52] Perelló-Oliver S., Muela-Molina C. (2012). La publicidad radiofónica en España y el perfil sociodemográfico de su audiencia [The radio advertising in Spain and the socio-demographic profile of his audience]. Palabra Clave.

[53] Polonsky M.J., Hyman M.R. (2007). A multiple stakeholder perspective on responsibility in advertising. J. Advert..

[54] Romero-Rodríguez L.M. (2020). Manual de Gestión de la Comunicación Corporativa.

[55] Thomas S.L., Bestman A., Pitt H., Cassidy R., McCarthy S., Nyemcsok C., Cowlishaw S., Daube M. (2018). Young people’s awareness of the timing and placement of gambling advertising on traditional and social media platforms: A study of 11–16-year-olds in Australia. Harm Reduct. J..

[56] Lopez-Gonzalez H., Estévez A., Jiménez-Murcia S., Griffiths M.D. (2018). Alcohol drinking and low nutritional value food eating behavior of sports bettors in gambling advertisements. Int. J. Ment. Health Addict..

[57] CNMC (Comisión Nacional de los Mercados y la Competencia) Resolución por la que se Requiere a Radio Popular, S.A (2016). Cope para que Cese la Emisión de Comunicaciones Comerciales de Bebidas Alcohólicas que no Respetan las Condiciones Establecidas en la Ley 7/201, de 31 de Marzo, General de la Comunicación Audiovisual. https://www.cnmc.es/sites/default/files/965670_7.pdf.

[58] Berelson B. (1952). Content Analysis in Communication Research.

[59] AIMC (Asociación para la Investigación de Medios de Comunicación) Resumen General del EGM (Estudio General de Medios) (2017). [EGM (Media General Study) General Report]. Febrero 2017 a Noviembre 2017..

[60] Cohen J. (1960). A coefficient of agreement for nominal scales. Educ. Psychol. Meas..

[61] Sánchez Carrión J.J. (1999). Manual de Análisis Estadístico de los Datos [Statistical Analysis of Data Manual].

[62] Muela-Molina C., Martín-Santana J.D., Reinares-Lara E. (2018). Journalists as radio advertising endorsers in news or talk radio stations. Journalism.

[63] Perelló-Oliver S., Muela-Molina C. (2019). Advertising self-regulation (ASR) in Spain. An analysis of complaints and resolutions. J. Mark. Commun..

